# Development of Theranostic ^177^Lu-Labeled Polymeric Nanoparticles (^177^Lu-PNPs) for the Treatment of Head and Neck Cancer

**DOI:** 10.1021/acsabm.5c00579

**Published:** 2025-05-29

**Authors:** Hsin-Hua Hsieh, Shih-Po Su, Yang-Hsiang Chan, Huihua Kenny Chiang, Yi-Jang Lee, Chun-Yi Wu

**Affiliations:** † Department of Biomedical Imaging and Radiological Sciences, National Yang Ming Chiao Tung University, Taipei 112, Taiwan; ‡ Genomics Research Center, Academia Sinica, Taipei 115, Taiwan; § Department of Biomedical Engineering, National Yang Ming Chiao Tung University, Taipei 112, Taiwan; ∥ Department of Applied Chemistry, National Yang Ming Chiao Tung University, Hsinchu 300, Taiwan; ⊥ Center for Emergent Functional Matter Science, National Yang Ming Chiao Tung University, Hsinchu 300, Taiwan; # Department of Medicinal and Applied Chemistry, Kaohsiung Medical University, Kaohsiung 807, Taiwan; ¶ Cancer and Immunology Research Center, National Yang Ming Chiao Tung University, Taipei 112, Taiwan

**Keywords:** ^177^Lu-labeled polymeric nanoparticles (^177^Lu-PNPs), head and neck cancer, NIR-II imaging, photothermal therapy, radiopharmaceutical therapy

## Abstract

This study presents the development of ^177^Lu-labeled polymeric nanoparticles (PNPs) for theranostic applications in head and neck cancer, utilizing both near-infrared II (NIR-II) and SPECT imaging for targeted delivery and monitoring. The PNPs were surface-modified with ethylenediamine and chelated with DTPA to enable ^177^Lu radiolabeling, achieving a radiochemical yield of 15.4% ± 3.2% and high purity (>95%). The radiolabeling process preserved the size distribution and optical properties of PNPs, facilitating their use in NIR-II imaging, which confirmed effective tumor delivery with peak uptake at 24 h postinjection. Photothermal therapy (PTT) combined with ^177^Lu-PNPs significantly enhanced tumor uptake and therapeutic efficacy, as shown by tumor growth suppression and extended survival in treated mice. Importantly, mice receiving the ^177^Lu-PNPs-PTT combination therapy exhibited minimal toxicity, as indicated by stable body weight and unaltered organ histology on H&E staining. These findings demonstrate the potential of ^177^Lu-PNPs as a safe and effective multifunctional platform for imaging-guided photothermal-radionuclide cancer therapy.

## Graphical abstract

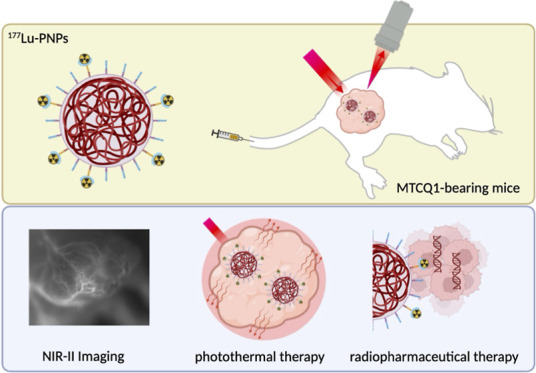

## Introduction

Head and neck squamous cell carcinoma (HNSCC), ranking sixth in cancer incidence, remains a medical challenge, especially for patients with recurrent tumors. Conventional treatments for HNSCC, such as surgery, radiotherapy, chemotherapy, and immunotherapy, often face limitations, and there is growing evidence that monotherapy may fall short of completely eradicating tumors.^1,2^ Identifying a practical therapeutic strategy is imperative to address this medical gap and better meet the complex needs of HNSCC patients.

Angiogenesis is a hallmark of HNSCC progression, contributing not only to tumor growth, invasion, and metastasis but also offering a valuable opportunity for imaging and therapeutic targeting.^3^ The abnormal and highly permeable vasculature characteristic of HNSCC promotes the enhanced permeability and retention (EPR) effect, enabling nanoparticles to preferentially accumulate in tumor tissues due to leaky vessels and impaired lymphatic drainage.^4^ This vascular abnormality also serves as a key imaging biomarker for assessing the disease progression and therapeutic response.

In this context, near-infrared-II (NIR-II) imaging (1000–1700 nm) has emerged as a powerful angiographic modality, offering significant advantages over conventional NIR imaging, including deep tissue penetration, reduced background autofluorescence, and high spatial resolution.^5^ These features enable noninvasive, high-contrast visualization of tumor vasculature and microenvironments, thereby guiding the precise delivery of nanoparticle-based therapeutics and supporting real-time monitoring of therapeutic outcomes in HNSCC. While NIR-II fluorescence may not penetrate deeply enough to allow whole-body imaging in human patients, it may nonetheless provide valuable insights during intraoperative or postoperative assessments. Moreover, in the preclinical setting, NIR-II imaging plays a critical role in accelerating the development and optimization of novel therapeutic strategies by enabling a detailed, high-resolution evaluation of drug delivery, tumor response, and vascular dynamics in animal models.

Photothermal therapy (PTT) involves materials proficient in converting laser light into thermal energy capable of accumulating in the tumor lesion. Upon exposure to laser light, the resulting localized heat has the potential to selectively eradicate tumors, minimizing damage to the surrounding healthy tissues. Previous studies have introduced multifunctional near-infrared-II (NIR-II) polymer semiconductor quantum dots designed to enhance photosensitivity at a specific light wavelength, displaying characteristics of mild PTT (approximately 45 °C).^6,7^ As localized heat accumulation induces vasodilation and increased permeability at the tumor site, the number of nanoparticles could be improved, amplifying the cytotoxic effects caused by the payload on the tumor. Several studies also indicated that mild PTT can induce immune cell death in maturing DC, stimulating T-cell activity, and enhancing NK cell response to trigger systemic immune responses.^8,9^

Lutetium-177 (^177^Lu) is currently gaining significant attention in tumor treatment as an attractive theranostic radionuclide. When incorporated into nanoparticles, ^177^Lu can simultaneously emit β^–^ radiation for therapeutic effects and release γ-rays for single-photon emission tomography (SPECT) imaging when retained in tumors. Numerous ^177^Lu-labeled nanocarriers have been employed for cancer treatment.^10–12^ Hsu et al. developed polymeric nanoparticles (PNPs) that are visible through a fluorescent imaging system and can be activated by a 793 nm laser for photothermal therapy.^13^ This study aims to label ^177^Lu onto PNPs and assess their therapeutic efficacy in combining β^–^ radiation and hyperthermia against head and neck cancer.

## Materials and Methods

### Materials

Ethyl-3-[3-(dimethylamino)­propyl]­carbodiimide (EDC), ethylenediamine, and 2-[4-(2-hydroxyethyl)-1-piperazinyl]­ethanesulfonic acid (HEPES) were purchased from Sigma-Aldrich Corp. (St. Louis, MO, USA). S-2-(4-Isothiocyanatobenzyl)-diethylenetriamine pentaacetic acid (*p*-SCN-Bn-DTPA) was purchased from Macrocyclics (Dallas, TX, USA). ^177^Lu-LuCl_3_ solution was obtained from Isotopia Molecular Imaging Ltd. (Petah Tikva, Israel). Formvar carbon films were purchased from Ted Pella, Inc. (Altadena, CA, USA). Cell culture dishes, plasticware, and Matrigel were purchased from Corning Inc. (Corning, NY, USA). Fetal bovine serum (FBS) and penicillin–streptomycin (PS) solution were purchased from HyClone (Logan, UT, USA). The Dulbecco’s modified Eagle’s medium (DMEM) powder was purchased from Gibco (Waltham, MA, USA). The experimental animals (CAnN.Cg-Foxn1^nu^/CrlNarl mice, 6 weeks old, male) were purchased from the National Laboratory Animal Center (NARLabs, Taipei, Taiwan).

### The Preparation of ^177^Lu-Labeled Polymeric Nanoparticles (^177^Lu-PNPs)

The PNPs utilized in this study were prepared using previously published methods^14^ and generously provided by Yang-Hsiang Chan at National Yang Ming Chiao Tung University, Hsinchu, Taiwan. These nanoparticles were formulated by co-assembling thermally activated delayed fluorescence (TADF) semiconducting polymers with mPEG-DSPE-2000 via high-intensity sonication. The absorption spectra of PNPs were assessed using a Biochrom Ultrospec 9000pc UV–visible spectrophotometer (Cambridge, UK). The surface modification of the PNPs is illustrated in Figure 1A. EDC (100 μg, 0.64 μmol) was dissolved in ddH_2_O and added to a PNP solution (200 μL, 5 mg/mL) to activate the carboxyl groups. After a 30 min reaction at room temperature (rt), ethylenediamine (9 μg, 0.15 μmol) was added to the reaction mixture and allowed to react for an additional 3.5 h. After removing unconjugated ethylenediamine through centrifugation at 5000*g* for 5 min, the resulting precipitates were collected and rinsed twice with ddH_2_O to obtain NH_2_-PNPs. Subsequently, the chelate *p*-SCN-Bn-DTPA (30 μg, 0.046 μmol) was added to the amine-modified PNPs, and the reaction mixture was left to react at 37 °C for 2 h. After removing unconjugated *p*-SCN-Bn-DTPA by centrifugation at 5000*g* for 5 min, the resulting precipitates were collected and rinsed twice with ddH_2_O to afford DTPA-modified PNPs (DTPA-PNPs). The zeta potential of PNPs was determined using a dynamic light scattering (DLS) scanner (#ZS90, Malvern, UK). Finally, ^177^Lu-LuCl_3_ was introduced into the HEPES solution (0.1 M, pH 4.5) containing DTPA-PNPs, and the reaction mixture was allowed to react at 40 °C for 2 h. After centrifugation at 5000*g* for 5 min, the resulting precipitates were collected and rinsed three times with ddH_2_O to yield the final product, ^177^Lu-PNPs. The labeling efficiency was assessed using radio-thin layer chromatography (radioTLC) on an instant TLC plate (ITLC, Merck, Darmstadt, Germany), with sodium citrate buffer (0.5 M, pH 5.0) as the mobile phase, and scanned with a radioTLC scanner (AR2000, Bioscan, CA, USA). The size and morphology of ^177^Lu-PNPs were visualized by transmission electron microscopy (TEM, JEM-2000EXII, Japan Electron Optics Laboratory Corp., Tokyo, Japan).

**Figure 1:**
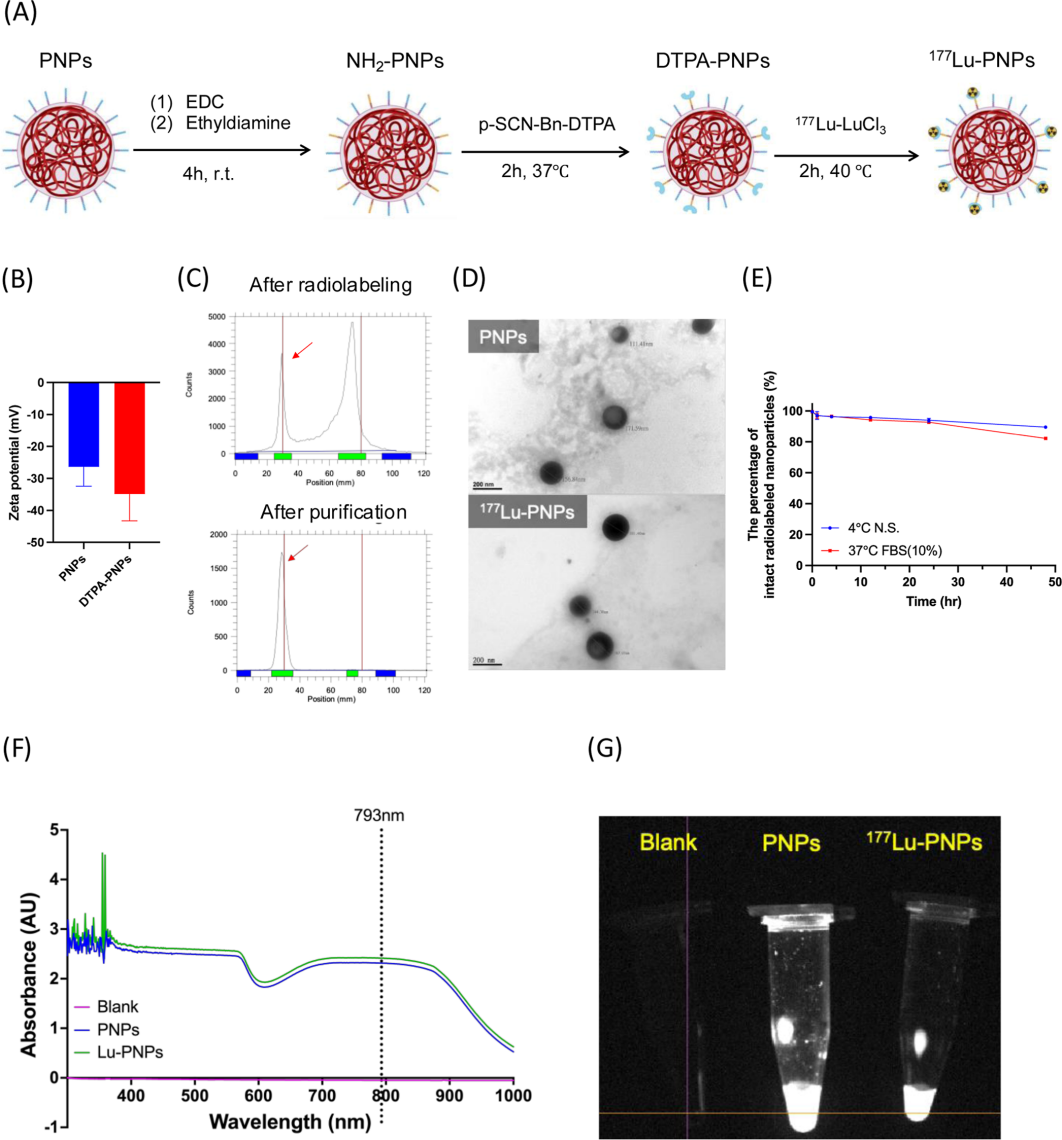
Physicochemical characteristics of ^177^Lu-PNPs. (A) Synthetic scheme of ^177^Lu-PNPs. (B) Zeta potential of PNPs and DTPA-PNPs. (C) Radio-thin layer chromatography analysis of ^177^Lu-PNPs. (D) Transmission electron microscopy imaging of PNPs and ^177^Lu-PNPs. (E) In vitro stability of ^177^Lu-PNPs. (F) UV spectrum analysis of PNPs and ^177^Lu-PNPs. (G) NIR-II imaging of PNPs and ^177^Lu-PNPs.

### The Stability of ^177^Lu-PNPs

The ^177^Lu-PNPs were incubated either in normal saline at 4 °C or in FBS at 37 °C for varying durations: 1, 4, 12, 24, or 48 h. To determine the percentage of intact ^177^Lu-PNPs at these specified time points, samples of ^177^Lu-PNPs were aspirated and subjected to radioTLC analysis.

### Near-Infrared-II (NIR-II) Imaging

NIR-II imaging was conducted using our custom-built system equipped with a 793 nm laser generator and an InGaAs camera, developed by Huihua Kenny Chiang at National Yang Ming Chiao Tung University, Taipei, Taiwan.^15^ When the tumor volume reached 100 ± 50 mm^3^, PNPs (10 mg/kg) were administered intravenously, and NIR-II imaging was performed at 5 and 30 min, as well as 24 and 48 h after injection.

### In Vitro and In Vivo Photothermal Conversion

A solution of PNPs in distilled water (5000 ppm) was placed into quartz cuvettes and subjected to a 793 nm laser at 1.0 W/cm^2^ for 15 min. The temperature elevation was measured using a digital thermometer (TES-1300, TES Electrical Electronic Corp., Taipei, Taiwan). The photothermal conversion efficiency (η) of PNPs was calculated using the equation reported by Ayala-Orozco et al.^16^ The photostability of PNPs was determined using previously published methods.^17^ For the in vivo photothermal conversion test, the tumors were exposed to a 793 nm NIR laser at a power of 1.0 W/cm^2^ for 3 min with a 2 min rest period, repeated twice, at 2 h after administering PNPs (10 mg/kg).

### Cell Incubation and Tumor Inoculation

The MTCQ-1 cells were cultured in Dulbecco’s modified Eagle’s medium (DMEM) supplemented with 10% FBS at 37 °C in a humidified atmosphere with 5% CO_2_. Approximately 1 × 10^6^ MTCQ-1 cells in 100 μL of serum-free medium were subcutaneously implanted into the right flank of male nude mice for tumor inoculation (NYCU IACUC 1110911). Experiments were initiated when the tumor size reached 100 ± 50 mm^3^.

### Animal SPECT/CT Imaging of ^177^Lu-PNPs

Animal SPECT/CT images were obtained at Chang Gung Memorial Hospital, Taoyuan City, Taiwan, using the nanoSPECT/CT imaging system (Mediso, Hungary). Static imaging was conducted for approximately 30 min at 2 and 24 h post-injection of around 9.25 MBq of ^177^Lu-PNPs. Standard uptake values (SUVs) for tumors and muscles were calculated using PMOD software (version 4.304). The tumor-to-muscle ratio (T/M) was utilized to account for specific tumor uptake and to eliminate individual differences.

### The Treatment Protocol

Upon the tumor size reaching 100 ± 50 mm^3^, the mice were randomly allocated into four groups (*n* ≥ 3 per group): control, PTT monotherapy (PNPs-PTT), ^177^Lu-PNP monotherapy, and combination treatment (^177^Lu-PNPs-PTT). The control group received an intravenous injection of normal saline on day 0, while the mice in ^177^Lu-PNP monotherapy and combination therapy groups were administered 7.4 MBq of ^177^Lu-PNPs. Additionally, mice in the PNPs-PTT and combination treatment group underwent a 5 min laser exposure (793 nm, 1 W/cm^2^) on day 0 at 2 h after injection of nanoparticles. Tumor size and body weight were monitored for 28 days following treatment, except for mice requiring euthanasia due to a tumor size ≥2000 mm^3^. A Kaplan–Meier plot was employed to illustrate survival outcomes.

### Western Blot

At the end of the treatment experiment, tumors were excised from mice in each group and treated with RIPA lysis buffer (Abcam, Cambridge, UK) at 4 °C for 30 min. The lysate was then centrifuged at 12,000*g* for 20 min at 4 °C, and the protein concentration was determined using the Pierce BCA Protein Assay Kit (Thermo Scientific, MA, US). Immunoblot analysis was performed with 8% and 12% SDS-PAGE according to the manufacturer’s instructions. Following a 1 h blocking step with 5% of bovine serum albumin (BSA, Sigma-Aldrich), the samples were incubated with p53 (GTX102965, GeneTex, 1:1000 dilution), BAX (50599-2-Ig, Proteintech, 1:1000 dilution), BCL-2 (GTX100064, GeneTex, 1:1000 dilution), caspase-3 (AB3623, Abcam, 1:2000 dilution), HSP70 (GTX639059, GeneTex, 1:2000 dilution), HSP90α (AB303516, Abcam, 1:2000 dilution), HSP90β (AB203085, Abcam, 1:2000 dilution), HMGB1 (EPR3507, Abcam, 1:2000 dilution), γH2AX (AB81299, Abcam, 1:2000 dilution), E-cadherin (GTX100443, GeneTex, 1:1000 dilution), N-cadherin (GTX127345, GeneTex, 1:1000 dilution), MMP9 (A2095, Abclonal, 1:1000 dilution), Vimentin (GTX100619, GeneTex, 1:5000 dilution), SNAI1 (Snail, GTX100754, GeneTex, 1:1000 dilution), or *anti*-β-actin (GTX109639, GeneTex, 1:5000 dilution). Following an overnight incubation at 4 °C, the membranes were treated with a 1:10,000 dilution of anti-horseradish peroxidase-conjugated anti-mouse/-rabbit secondary antibody (#32430/#31460, Thermo Scientific). The intensity of specific bands was measured using a Trident femto Western HRP Substrate kit (GTX14698, GeneTex), and images were captured using an ImageQuant LAS 4000 (GE Healthcare Bio-Sciences Corp., USA). Quantitative analysis was performed using ImageJ software (version 1.53k).

### H&E Staining

At the end of the treatment experiment, tumors, kidneys, livers, and spleens were excised from mice in each group and fixed with 4% paraformaldehyde at 4 °C for 1 day. Following fixation, the organs were embedded in paraffin, and 5 μm thick slices were prepared. These slices underwent a sequential process involving immersion in xylene for 30 min, followed by absolute ethanol, 85% ethanol, and 75% ethanol for every 5 min at rt. Hematoxylin (no. 200228, Muto Pure Chemical Co., Ltd., Tokyo, Japan) was stained for 1 min and rinsed with water for 5 min. Subsequently, eosin (#200302, Muto Pure Chemical Co., Ltd., Tokyo, Japan) was stained for 1 min, rinsed with water, and dehydrated with gradient ethanol. The slides were scanned using a panoramic microscope (Axioscan7, Zeiss, Oberkochen, Germany).

### Statistical Analyses

Statistical analyses were conducted using unpaired *t* tests and two-way ANOVA in Prism 10.1.0, and the results were presented as the mean ± standard deviation. Survival rates between different groups were compared using the Kaplan–Meier method with the log-rank test. A *p*-value less than 0.05 (*p* < 0.05) was considered statistically significant.

## Results

### The Preparation and Characteristics of ^177^Lu-PNPs

The zeta potential of DTPA-PNPs (−34.9 ± 8.34 mV) increased after chelate modification (Figure 1B), indicating the successful modification of DTPA chelates. The labeling efficiency of ^177^Lu-PNPs was approximately 28.8% ± 4.3%, as depicted in Figure 1C. Notably, the radiochemical purity of ^177^Lu-PNPs could still be achieved at >95% following purification with a radiochemical yield of 15.4 ± 3.2%. TEM analysis revealed that the size of the ^177^Lu-PNPs measured approximately 167.4 ± 17.4 nm and indicated that the radiolabeling process had no detrimental impact on the size and morphology (Figure 1D). Stability assays revealed that the percentage of intact ^177^Lu-PNPs remained over 90% in both normal saline and FBS for up to 24 h (Figure 1E). Moreover, UV spectrum analysis and NIR-II imaging found that radiolabeling did not significantly alter the light absorption and emission properties of PNPs (Figure 1F,G).

### Photothermal Conversion Characteristics of PNPs

After 8 min of laser irradiation, the temperature of the ddH_2_O control increased by only 0.8 °C, while that of vials containing various concentrations of PNPs elevated in a range of 16.0–29.6 °C, demonstrating a concentration-dependent manner (Figure 2A). Upon turning off the laser, the temperature-cooling curve of PNPs after laser irradiation is illustrated in Figure 2B. Based on these results, the calculated τs and photothermal conversion efficiency (η) were 223.9 s and 81.1%, respectively (Figure 2C). The reproducible and constant temperature increase indicated the superior photothermal stability of PNPs (Figure 2D). In vivo thermal images of mice in each group are presented in Figure 2E. A similar temperature increase was observed in the PNP- and ^177^Lu-PNP-injected groups. The mean temperature elevations of the controls, PNP-injected mice, and ^177^Lu-PNP-injected mice were 1.5 ± 0.4 °C, 6.9 ± 1.6 °C, and 8.6 ± 0.8 °C, respectively, suggesting that the radiolabeling did not compromise the photothermal conversion ability of PNPs.

**Figure 2:**
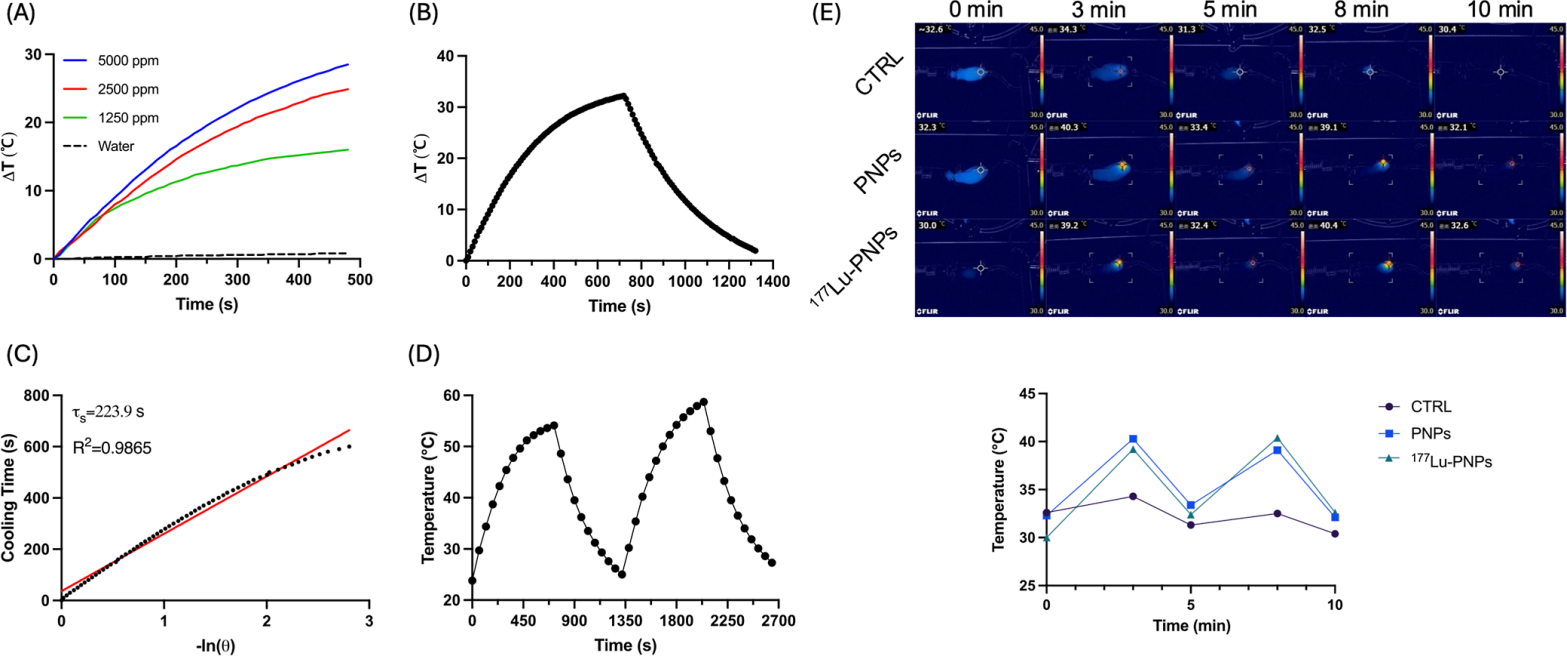
Photothermal properties of PNPs. (A) Temperature changes of PNPs at various concentrations during 793 nm laser exposure. (B) Heating–cooling curve of PNPs at a concentration of 5000 ppm. (C) Time constant linear curve of PNPs (5000 ppm). (D) Temperature changes of PNPs (5000 ppm) during repeated laser exposures. (E) Temperature changes on the tumor surface during laser exposures.

### In Vivo Distribution of ^177^Lu-PNPs

Quantitative analysis of NIR-II imaging revealed an initial increase in tumor uptake for both mice injected with PNPs and ^177^Lu-PNPs, reaching a peak at 24 h post-injection (p.i.) before experiencing a slight decline (Figure 3A). During the early time points, the fluorescent signals within the tumor served as indicators of blood vessel density (Figure 3B). Moreover, at 2 h p.i., delivering laser exposure to the mice injected with ^177^Lu-PNPs significantly enhanced tumor uptake compared to those that did not receive mild PTT (Figure 3C). Representative microSPECT/CT images of tumor-bearing mice that received intravenous injections of ^177^Lu-PNPs are shown in Figure 3D. The average tumor-to-muscle ratio of controls was 1.3 ± 0.4, while that of mice exposed to mild PTT was significantly elevated to 3.2 ± 0.2, consistent with the observation in NIR-II imaging.

**Figure 3:**
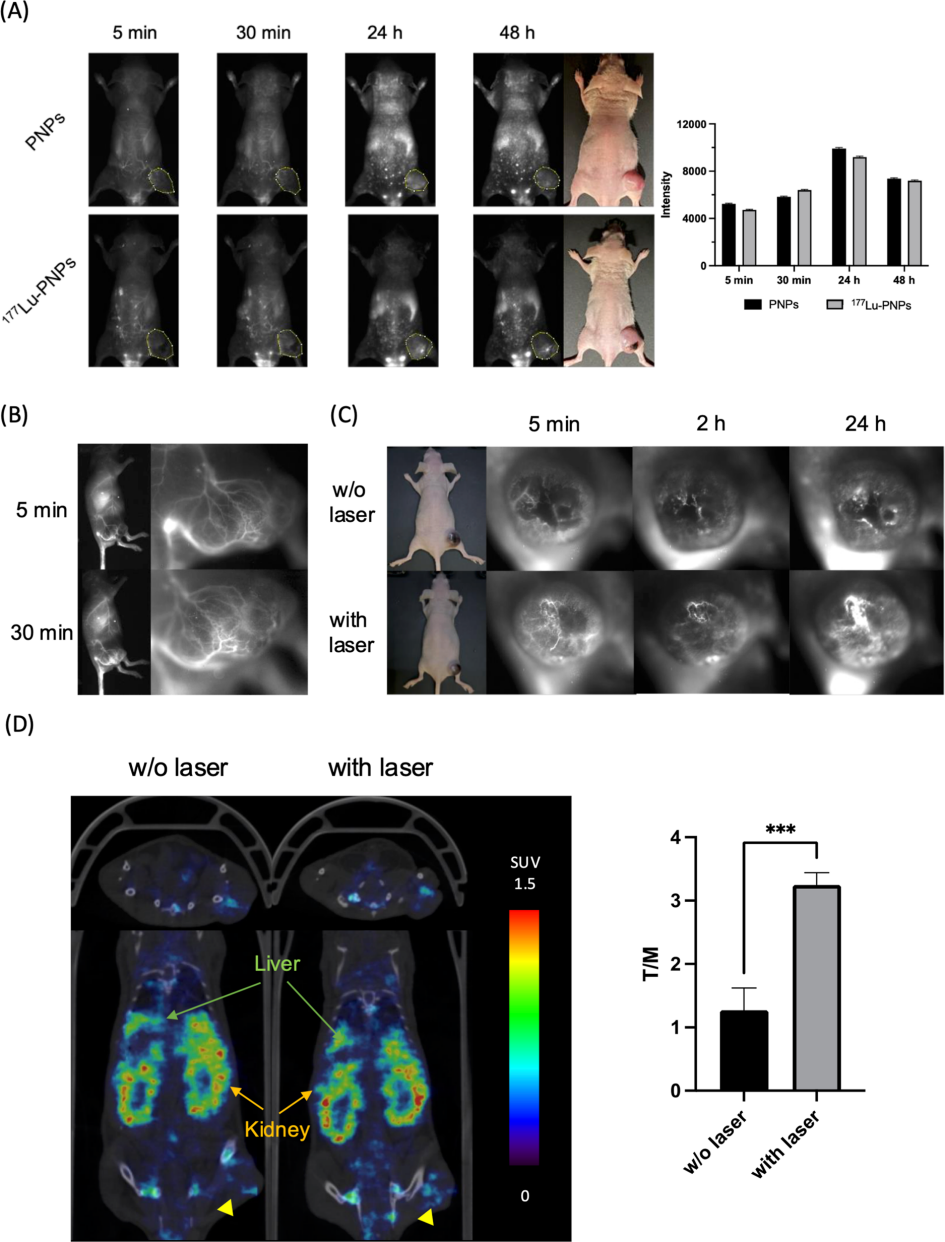
NIR-II imaging and SPECT imaging of PNPs and ^177^Lu-PNPs. (A) The NIR-II imaging and quantitative analysis of MTCQ-1 tumor-bearing mice injected with PNPs and ^177^Lu-PNPs. (B) Focused NIR-II imaging of tumor lesion at 5 and 50 min after injection of PNPs. (C) The tumor accumulation of ^177^Lu-PNPs with and without laser exposure. (D) The SPECT imaging and quantitative analysis of tumor-bearing mice injected with ^177^Lu-PNPs. Arrowheads indicate tumors.

### The Therapeutic Efficacy of Combination Therapy

Figure 4A outlines the treatment protocol employed in this study. Control mice treated with normal saline met euthanasia criteria at around day 17. PNPs-PTT or ^177^Lu-PNP monotherapy displayed initial antitumoral effects, but tumors continued to proliferate around day 10. The combination therapy group exhibited the most apparent tumor retardation effect (Figure 4B). In addition, combination therapy successfully prolonged the survival time without significantly affecting the body weight (Figure 4C). H&E staining revealed that tumors in the combination therapy group had the lowest nuclear-cytoplasmic ratio, aligning with findings on therapeutic efficacy (Figure 4D). Additionally, there was no significant difference in histological morphology among organs with high ^177^Lu-PNP accumulation in each group (Figure 4D).

**Figure 4:**
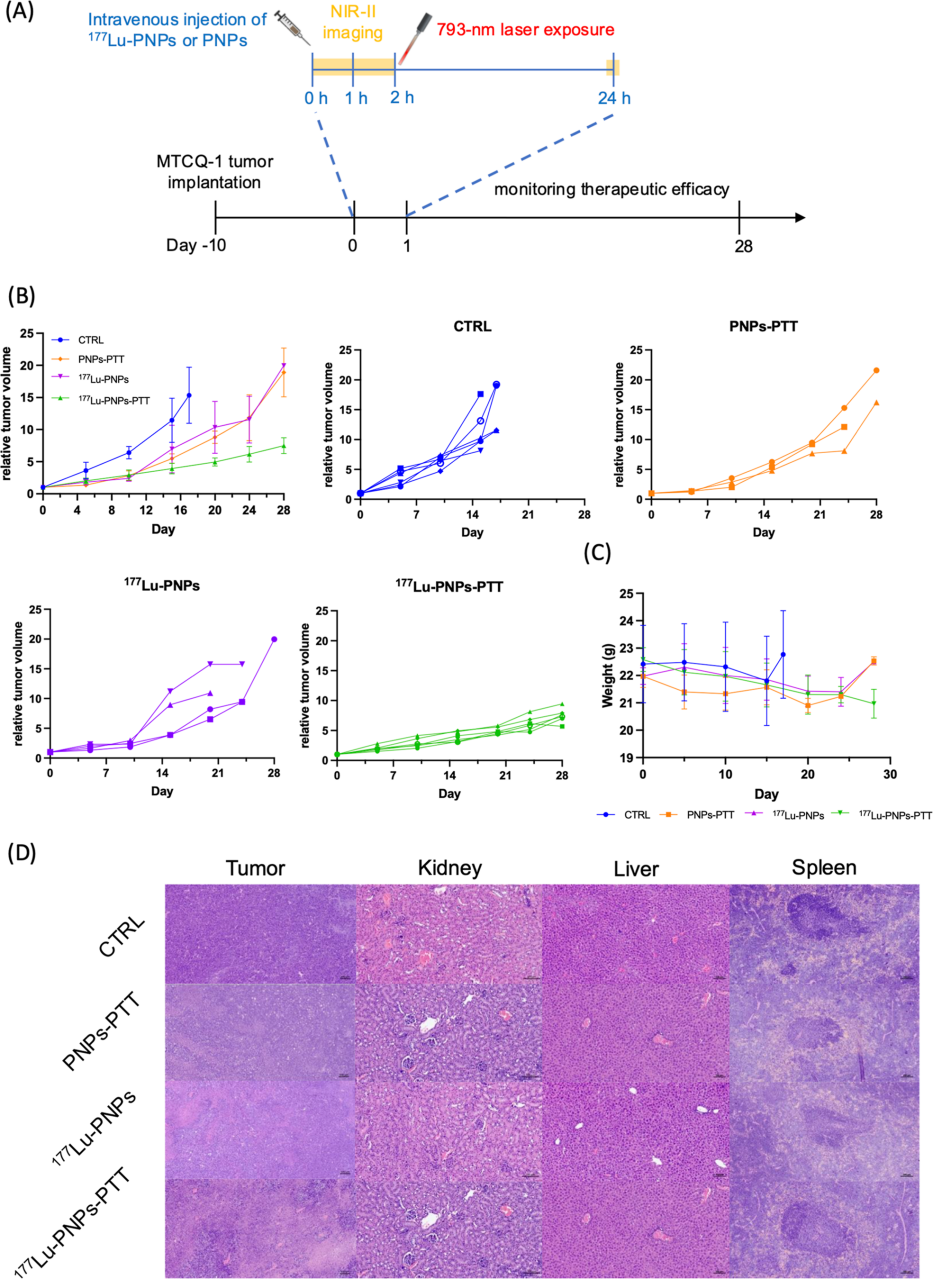
Therapeutic effectiveness of monotherapy and combination therapy. (A) The treatment timeline. (B) Relative tumor volume of mice. (C) Body weight of mice in each group. (D) Histological analysis for tumors and organs from each group.

### Effects of Combination Therapy on the Tumor Microenvironment

Following combination treatment, an upregulation in p53 expression was observed, resulting in an increased BAX-to-BCL-2 ratio and subsequent activation of the caspase-3-mediated apoptosis pathway (Figure 5A). Specifically, in the group receiving combined ^177^Lu-PNP and PTT treatment, surviving tumor cells exhibited marked activation of caspase-3, indicating the induction of apoptosis (Figure 5A). Notably, higher levels of HSP70, HSP90α, and HSP90β were also noticed in the residual tumor cells (Figure 5B), indicating the impact of PTT. Additionally, the upregulated high mobility group box 1 (HMGB1) and γH2AX in tumors receiving combination therapy confirmed radiation-induced damage (Figure 5B). However, the surviving tumor cells exhibited upregulation of the transcription factor Snail, accompanied by persistent elevation of N-cadherin, MMP9, and vimentin. This is indicative of an increased migratory potential (Figure 5C).

**Figure 5:**
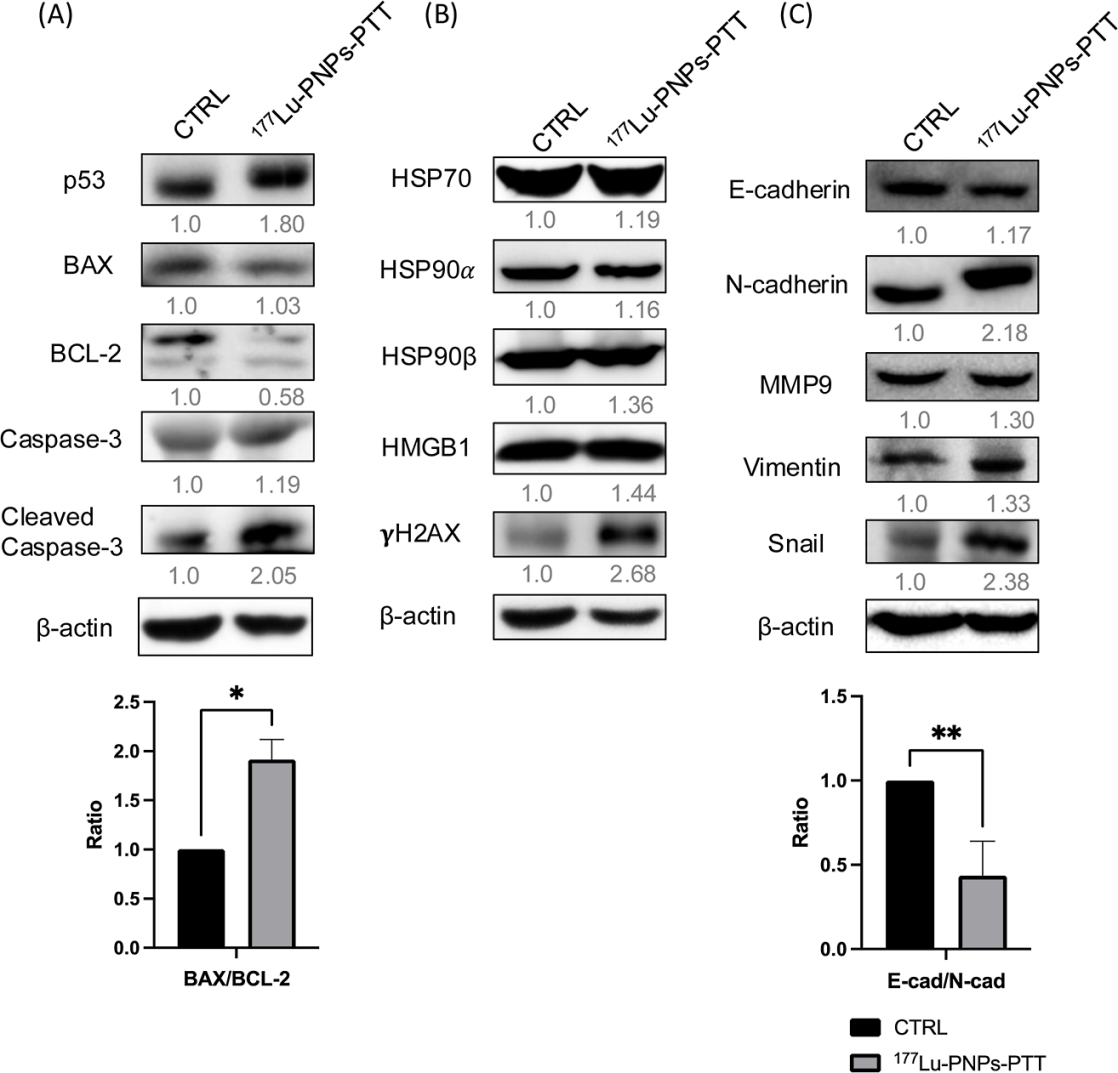
Western blot analysis of residual tumors after combination therapy. (A) Apoptosis-related proteins, (B) heat damage and radiation damage-associated proteins, and (C) the epithelial–mesenchymal transition pathway.

## Discussion

Theranostic agents have garnered significant attention for their potential to utilize noninvasive imaging for predicting therapeutic efficacy, aiding in patient selection before treatment.^18^ Therefore, we labeled the therapeutic radioisotope, ^177^Lu, onto multifunctional nanoparticles, enhancing their anti-tumoral therapy capabilities in this study. Although the radiochemical purity of ^177^Lu-PNPs exceeded 95% after purification, the relatively low radiolabeling efficiency limited the radiochemical yield (Figure 1C). The relatively low radiolabeling efficiency observed in our system can likely be attributed to the structural characteristics of the PNPs. Specifically, the carboxyl groups intended for chelate conjugation appeared to be encapsulated within the nanoparticle shell rather than being readily exposed on the surface. This limited surface accessibility reduces the number of available amine groups for effective chelate modification, thereby leading to suboptimal chelate modification. Furthermore, previous studies have demonstrated that the elevated temperatures (approximately 100 °C) can significantly enhance the radiolabeling efficiency of radiometals such as lutetium-177.^19^ However, our PNPs exhibit notable thermal sensitivity, making them susceptible to structural collapse or degradation when exposed to elevated temperatures. This characteristic limits the applicability of high-temperature labeling protocols, thereby constraining our ability to improve radiolabeling performance through conventional thermal activation. Despite these limitations, it is important to note that the radiolabeling process did not adversely affect the key functional properties of the PNPs. As shown in Figures 1F,G and 2A,D, both the NIR-II fluorescence emission and photothermal conversion capabilities remained intact post-labeling. This preservation of multifunctionality supports the continued use of these PNPs in integrated applications, including NIR-II imaging, radiopharmaceutical therapy, and photothermal therapy, highlighting their potential as versatile theranostic platform.

To address the issue of fluorescent light penetration, our developed PNPs exhibit fluorescent emission in the NIR-II window, facilitating the detection of deep-seated tumors (Figure 3A). Additionally, PNPs can serve as a contrast agent to perform a “tumor angiogram” after intravenous injection within a short-term period (Figure 3B). The fluorescent signals in the tumor reflect the tumor blood vessel density, a critical factor influencing the enhanced permeability and retention (EPR) effect of nanoparticles and the treatment response of radiolabeled nanoparticles.^20^ As anticipated, limited therapeutic efficacy was observed in hypovascular tumors treated with ^177^Lu-PNPs (Figure S2). Therefore, NIR-II imaging holds the promise that before radiopharmaceutical therapy it can guide physicians in the optimal management of patients.

Unlike heat ablation therapy, PTT generates concentrated heat within the tumor without significant outward spreading due to the specific accumulation of photothermal conversion agents in the lesion. Huang et al. developed aggregation-induced emission (AIE) dots with photothermal conversion capability and found the surface temperature of 4T1 tumors increased to exceed 50 °C after a 10 min exposure to an 808 nm laser with a power of 0.8 W/cm^2^. However, when these dots were labeled with ^177^Lu, there was no significant difference in tumor suppression ability between tumor-bearing mice treated with AIE dots and those treated with ^177^Lu-labeled AIE dots.^21^

In contrast, the PNPs used in the present study primarily induced a mild thermal effect, raising the tumor temperature by approximately 10 °C in the tumor. This mild hyperthermia enhanced vascular permeability, as evidenced by a 2.5-fold increase in the T/M ratio in microSPECT imaging (Figure 3D). Importantly, the PNPs exhibited excellent photostability under repeated laser irradiation with no significant photobleaching or photofatigue observed during the imaging or treatment window. Regarding metabolic stability, PNPs are known to be gradually cleared via the hepatobiliary and reticuloendothelial systems.^22^ Although partial metabolic degradation may occur over time, the short interval between nanoparticle administration and photothermal treatment (typically within 24 h) likely minimizes any substantial impact on photothermal performance. In our study, NIR-II signals remained stable during this period, and therapeutic efficacy was preserved, suggesting that in vivo nanoparticle transformation did not significantly affect the photomechanism within the experimental time frame.

Regarding therapeutic efficacy, although both the PTT and ^177^Lu-PNP monotherapy groups exhibited modest inhibition of tumor growth during the early phase of treatment, all tumors in these groups progressed to meet the euthanasia criteria within 28 days (Figure 4B). In contrast, the combination therapy group showed a markedly reduced tumor proliferation rate, as reflected by the gentler slope of the tumor volume curve. Notably, no mice in the combination group required euthanasia during the 28 day observation period (Figure 4B), underscoring the superior efficacy of the combined treatment approach.

^177^Lu-PNP radiopharmaceutical therapy induced higher expression of γH2AX, indicating that potent DNA damage occurred, and provoked the apoptotic pathways in the tumor, as evidenced by the elevated BAX/BCL-2 ratio (Figure 5A,B). Additionally, for the effect of PTT, we observed increased release of damage-associated molecular patterns (DAMPs), such as HMGB1, which would recruit antigen-presenting cells to the tumor lesion, thereby facilitating immunogenic cell death in tumors (Figure 5B). However, its role is controversial.^23,24^ Dong et al. indicated that the overexpression of HMGB1 would enhance radioresistance and migration in esophageal squamous cell carcinoma.^25^ Mukhopadhya et al. indicated that hyperthermia could provoke the release of HSPs, which are associated with dendritic cell maturation.^26^ Although we noticed this phenomenon, HSPs also have been implicated in inducing extracellular matrix (ECM) remodeling, EMT, and resistance of apoptosis, which may increase tumor aggressiveness.^27,28^ The multifaceted roles of these factors urge further studies to determine the detailed mechanism of RPT plus PTT. This complexity may also explain why combination therapy can effectively control tumor growth but not completely eradicate the tumor. Yan et al. revealed N-cadherin expression had a significantly opposite overall survival and disease-free survival rate, which serves as a novel prognostic predictor for CRC.^29^ In addition, the cadherin switch from E to N, represented as the E-cadherin-to-N-cadherin ratio, mediated cancer progression via TGF-β-induced epithelial-to-mesenchymal transition.^30,31^

While this study demonstrated promising results, several limitations should be acknowledged. First, as this pilot study aimed at assessing the feasibility of combining mild PTT with radiopharmaceutical therapy using our developed multifunctional nanoparticles, the dosage and treatment timing were not optimized. Consequently, complete remission was not observed in mice treated with a combination therapy. Additionally, Western blot assays revealed that residual tumor cells displayed increased aggressiveness and metastatic potential, underscoring the need to address this issue in future investigations. Second, the study lacks a detailed elucidation of the mechanisms underlying how mild PTT enhances the therapeutic efficacy of radiopharmaceutical therapy. We hypothesize that the elevated temperature induced by PTT in the tumor enhances the permeability, facilitating the transportation of more radiolabeled nanoparticles to tumor lesions and subsequent tumor cell death. Investigating this relationship further may lead to even greater synergistic effects and improved therapeutic efficacy.

## Conclusions

In this study, we have successfully developed theranostic ^177^Lu-PNPs, which can serve as multifunctional platform versatile agents for angiography, photothermal therapy, and radiopharmaceutical therapy. We also found that a mild photothermal treatment can significantly enhance the therapeutic efficacy of ^177^Lu-PNPs. These findings underscore the promise of ^177^Lu-PNPs as cutting-edge theranostic tools in the management of cancer.

## Supplementary Material



## Data Availability

The authors will supply the relevant data in response to reasonable requests.

## Notes

Correlation between ^177^Lu-PNP concentration and NIR-II fluorescence intensity and NIR-II imaging in tumors as an indicator for selecting candidates for ^177^Lu-PNP treatment (PDF)
