# Supplementary material for: Development of Theranostic 177Lu-Labeled Polymeric Nanoparticles (177Lu-PNPs) for the Treatment of Head and Neck Cancer
Source: ACS Appl Bio Mater. 2025 May 29;8(6):5266–75. doi: 10.1021/acsabm.5c00579 (PMC12175126; doi:10.1021/acsabm.5c00579)
Supplement: Supplementary file 1 [file mt5c00579_si_001.pdf]

Supporting Information

Development of Theranostic ^{177}Lu-labeled Polymeric Nanoparticles (^{177}Lu-PNPs) for the Treatment of Head and Neck Cancer

Hsin-Hua Hsieh[a], Shih-Po Su[b], Yang-Hsiang Chan[c,d,e], HuiHua Kenny Chiang[b], Yi-Jang Lee[a], Chun-Yi Wu[*,1]

[a] *Department of Biomedical Imaging and Radiological Sciences, National Yang Ming Chiao Tung University, Taipei 112, Taiwan*

[b] *Department of Biomedical Engineering, National Yang Ming Chiao Tung University, Taipei Taiwan 112, Taiwan*

[c] *Department of Applied Chemistry, National Yang Ming Chiao Tung University, Hsinchu 300, Taiwan*

[d] *Center for Emergent Functional Matter Science, National Yang Ming Chiao Tung University, Hsinchu 300, Taiwan*

[e] *Department of Medicinal and Applied Chemistry, Kaohsiung Medical University, Kaohsiung 807, Taiwan*

*Correspondence: Chun-Yi Wu, Ph.D., Department of Biomedical Imaging and Radiological Sciences, National Yang Ming Chiao Tung University, Taipei, Taiwan.

No. 155, Sec. 2, Li-Nong St., Beitou, Taipei 112, Taiwan.

E-mail: chunyiwu@nycu.edu.tw

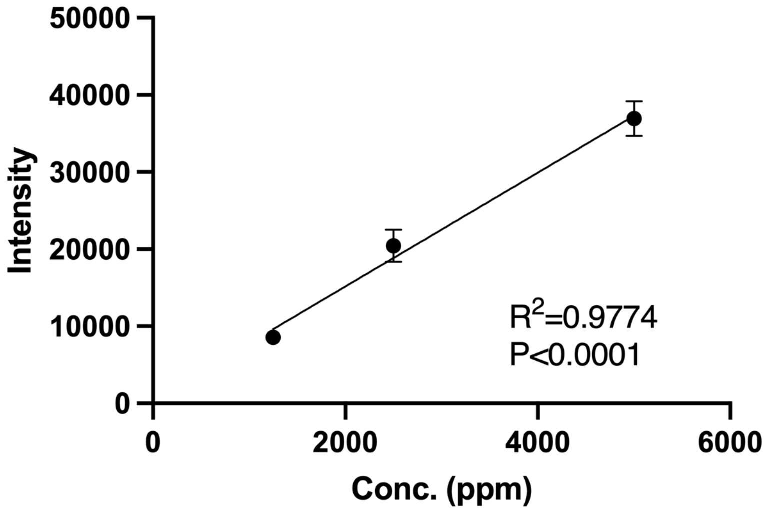

Fig S1. NIR-II fluorescence intensity correlated with ^{177}Lu-PNP concentration in a dose-dependent manner. Increasing concentrations of ^{177}Lu-PNPs result in proportional enhancement of the NIR-II signal, indicating a quantitative relationship between nanoparticle accumulation and imaging intensity.

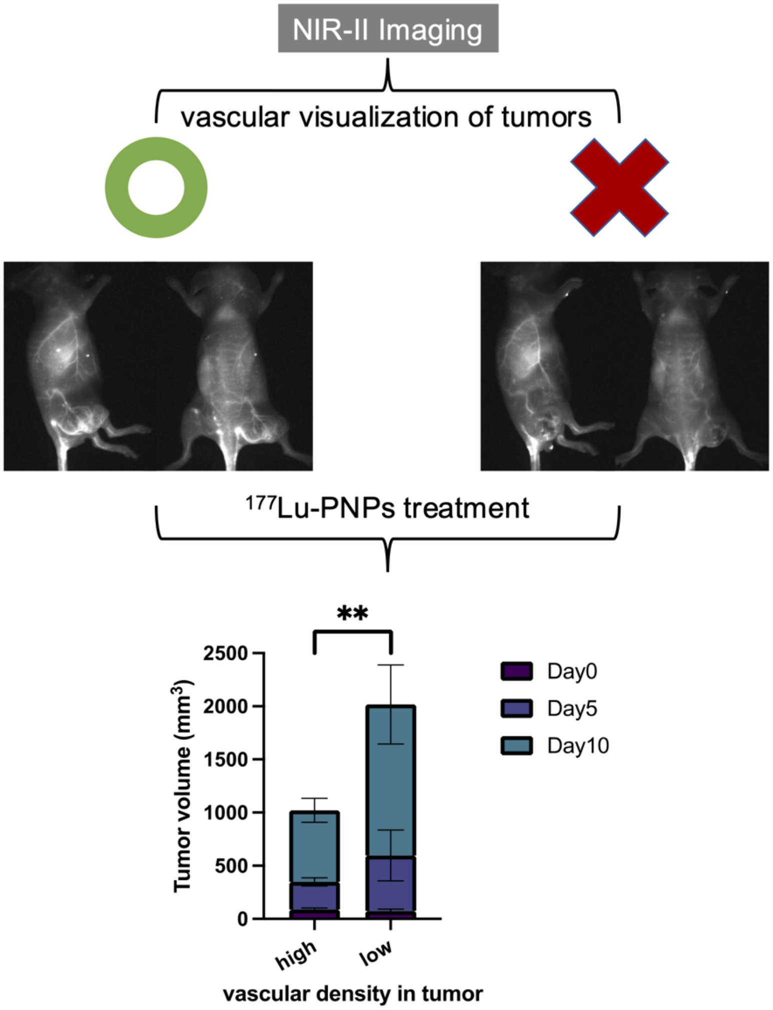

Fig S2. The blood vessel density assessed via NIR-II imaging in tumors can serve as an indicator for selecting candidates for ^{177}Lu-PNPs treatment. Tumors with relatively low blood vessel density are linked to poor treatment response.
